# *Paraphlomis kuankuoshuiensis* (Lamiaceae), a new species from the limestone areas of northern Guizhou, China

**DOI:** 10.3897/phytokeys.139.47055

**Published:** 2020-01-15

**Authors:** Ren-Bo Zhang, Tan Deng, Quan-Li Dou, Ruo-Xun Wei, Lin He, Chong-Bo Ma, Sheng Zhao, Shun Hu

**Affiliations:** 1 Department of Biology, Zunyi Normal College, Zunyi, Guizhou 563002, China Zunyi Normal College Zunyi China

**Keywords:** Guizhou, karst, limestone flora, new taxon, Paraphlomideae, *Paraphlomis kuankuoshuiensis*

## Abstract

*Paraphlomis kuankuoshuiensis* (Lamiaceae), a new species found in the limestone areas of northern Guizhou, China, is described and illustrated in this paper. Based on its tubular-campanulate calyx, this taxon should be a member of sect. Paraphlomis Prain. The new species resembles *P. patentisetulosa* C.Y. Wu & H. W. Li, *P. hispida* C.Y. Wu, and *P. hirsutissima* C.Y. Wu & H.W. Li, but differs from these three taxa in the following aspects: the stems are very short (<7 cm), with one or two short internodes, giving the impression of having a tuft of basal leaves; it has sparsely setose hairs on the outer surface of the calyces and short fruiting calyces. The florescence, fruit period, habitat, and the geographical distribution of *P. kuankuoshuiensis* are also quite different from the three closely related species.

## Introduction

*Paraphlomis* (Prain) Prain is a genus of about 24 species in Lamiaceae (Ko et al. 2014), of which 23 *Paraphlomis* species and seven varieties were recorded from China (China Botanical Flora Editorial Committee 1977, Li and Hedge 1994, Xiang et al. 2010). The genus is characterized by erect galeate corollas with longer upper lips than lower lips; rounded and bearded upper corolla lips, five-toothed calyces, and two-cleft, sub-equal style apices (Ko et al. 2014).

*Paraphlomis* was separated from *Phlomis* by Prain in 1901 (Azizian and Moore 1982) and was supported as an independent genus (Pan et al. 2009). Scheen et al. (2010) placed the genus *Paraphlomis* in the tribe Lamioideae and they suggested that its phylogenetic position is uncertain although their analyses do place two accessions of *Paraphlomis* in the vicinity of *Phlomis*. Based on the DNA sequence data from chloroplast regions, Bendiksby et al. (2011) established a new tribe, Paraphlomideae which includes three genera: *Paraphlomis* Prain, *Matsumurella* Makino, and *Ajugoides* Makino. Results from Li et al. (2016) also supported the circumscription of the tribe.

In recent years, a small number of new *Paraphlomis* taxa were reported across mainland China, including *P. breviflora* B.Y. Ding, Y.L. Xu et Z.H. Chen (Ding et al. 2019) in the Zhejiang Province; P. javanica (Blume) Prain var. pteropoda D. Fang & K.J. Yan and P. javanica (Blume) Prain var. angustifolia C.Y. Wu & H.W. Li f. albinervia D. Fang & K.J. Yan (Yan and Fang 2009), are both from the Guangxi Province. In addition, Xiang et al. (2016) treated two varieties of *Paraphlomis* species as new synonyms: P. javanica var. pteropoda D. Fang et K. J. Yan as P. javanica (Blume) Prain var. javanica and P. javanica var. angustifolia f. albinervia D. Fang et K. J. Yan as P. javanica var. angustifolia (C. Y. Wu) C. Y. Wu et H. W. Li, respectively.

During fieldwork, a new species of *Paraphlomis* was discovered in Kuankuoshui National Natural Reserve, Suiyang County, Guizhou Province, China. Based on its tubular-campanulate calyces, the new species was placed in Sect. Paraphlomis. This species has bristled tubular-campanulate calyces, conspicuous calyx teeth, and oblong-elliptic and hairy leaves which resemble *P. patentisetulosa* C.Y. Wu et H.W. Li, *P. hispida* C.Y. Wu and *P. hirsutissima* C.Y. Wu & H.W. Li. However, the new species differs from these three taxa in a variety of ways. For instance, it has very short stems (< 7 cm), with one or two short internodes, giving the impression of having a tuft of basal leaves; it has sparsely setose hairs on the outer surface of the calyx and short fruiting calyces. Morphological characteristics indicate that this species differs from the above mentioned *Paraphlomis* species and should be considered a new species in this genus.

## Materials and methods

All morphological characteristics were measured using dissecting microscopes. The flowering and fruiting specimens of the potential new species were checked at ZY (Thiers 2019). Three specimens of *P. patentisetulosa* were observed at IBSC and their collecting numbers were 37835 (Type), 163476, and 12441. One specimen of *P. hispida* (92449) was observed at IBK. The following traits (some of them not described in "Flora of China") were carefully surveyed: indumentum, length of the corolla, shape and length of the bracteoles, (fruiting) calyx tubes and teeth, and nutlets (92449 has no nutlet).

Digital specimens, including 33 taxa belonging to 19 *Paraphlomis* species (Table S1), were checked at AU, BH, BNU, FJFC, GXMG, IBK, JIU, JJF, KUN, NAS, PE, and SM herbaria through NSII platform (http://www.nsii.org.cn/2017/home.php), with the additional consultation of online databases, including the Plant Photo Bank of China (http://ppbc.iplant.cn/), Chinese Field Herbarium (http://www.cfh.ac.cn/), and Global Plants (http://plants.jstor.org/).

## Results

### Paraphlomis kuankuoshuiensis

Taxon classificationPlantaeLamialesLamiaceae

R.B.Zhang, D.Tan & C.B.Ma sp. nov.

7527396F-9D02-58C8-9773-223C61A1E42F

urn:lsid:ipni.org:names:77204425-1

Figs 1 , 2

#### Diagnosis.

*Paraphlomis kuankuoshuiensis* can be distinguished from the morphologically similar species *P. patentisetulosa, P. hispida*, and *P. hirsutissima* by its very short stem (< 7 cm) with one or two short internodes (giving the impression of having a tuft of basal leaves). The three closely related species have stems longer than 15 cm and more internodes. The new species has sparsely setose hairs on the outer surface of the calyces (vs. finely or densely) and short fruiting calyces (5-6 cm vs. 7 cm, 8-9 cm, and to 11 cm). There are some other diagnostic characters between the new species and its three closely related species (Table 1). The flowering from July to August and fruiting from August to September are quite different from the three species (vs. fruiting from November to January). It grows on bare steep rocks, which is a distinctive habitat. The new species distributes in Guizhou Province and is far away from the other three species (Fig. 3).

**Table 1.** Comparing the diagnostics of *Paraphlomis kuankuoshuiensis* sp. nov., *P. patentisetulosa*, *P. hispida*, and *P. hirsutissima*.

**Traits**		***P. kuankuoshuiensis* sp. nov.**	***P. patentisetulosa***	***P. hispida***	***P. hirsutissima***
Stems	Height (cm)	**2–7**	15–25	Ca. 60	> 20
	Habit	**Erect and tufted**	Ascending	Slightly ascending	Flexuous
	Habitat	**Steep rock surface beside stream**	Beside stream	In tropical forests or thickets	In gravels below tropical forests
Leaf blades (cm)		10–**37** × 3–8	5.5–14.5 × 2.5–7	3–20 × 1.8–11.5	5.5–13 × 2–5
Fruiting calyces	Shape	Tubular-campanulate	Tubular-campanulate	Tubular-campanulate	**Tubular**
	Length (mm)	**5–6**	To 11	Ca. 7	8–9
	Hairs	**Sparsely** bristly, glabrous inside	Finely bristly outside	Densely hispid, glabrous inside	?
	Tooth length (mm)	Ca. 2	Ca. 3	**Ca. 5**	Ca. 2
	Tooth direction	Erect	Erect	Erect	**Reflexed**
Nutlet apices		Truncate	**Rounded**	Truncate	?
Fl.		**Jul–Aug**	?	?	?
Fr.		**Aug–Sep**	Nov	Nov–Jan	Jan
Distributed province in China		**Guizhou**	Guangdong	Yunnan [Vietnam]	Yunnan

Note: question mark (?) indicates that that character is not described in the references.

**Figure 1.**
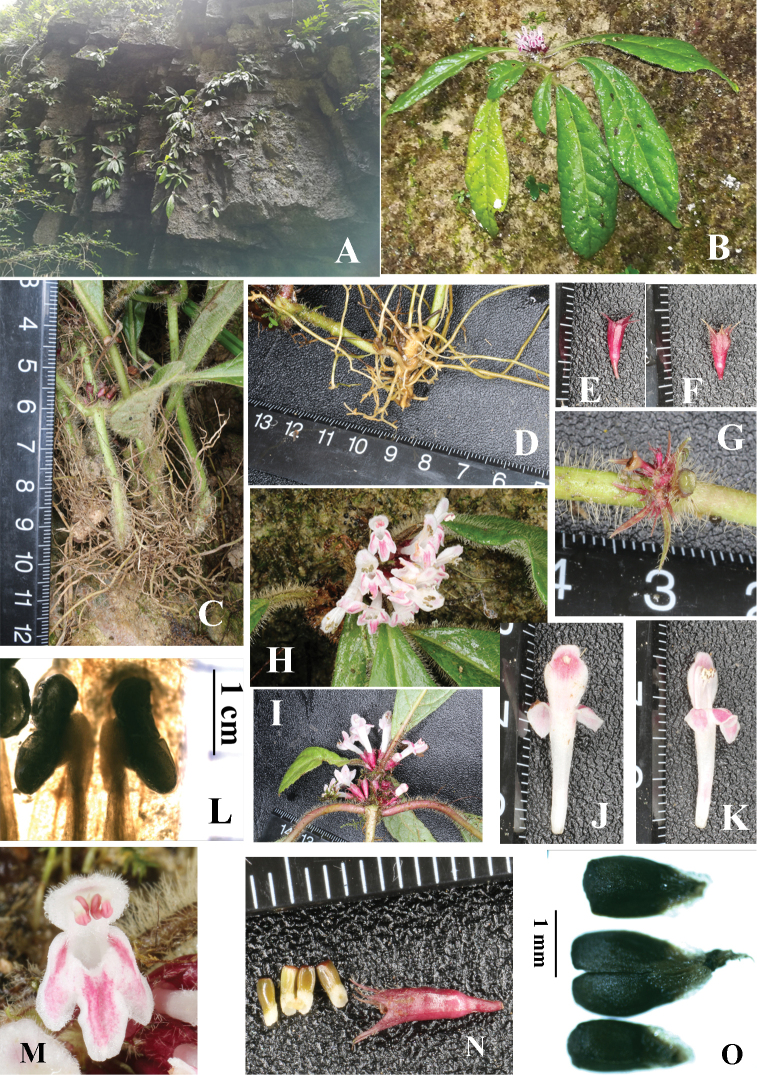
*Paraphlomis kuankuoshuiensis* spe. nov. **A** natural habitat **B** flowering plant **C** short and tufted stems **D** rhizome **E–F** calyces **G** bracteoles **H** frontal view of verticillaster **I** lateral view of verticillaster **J–K** corolla **L** anthers **M** front view of corolla **N** fresh nutlets and fruiting calyx **O** dried nutlets. Photographed by Ren-Bo Zhang.

**Figure 2.**
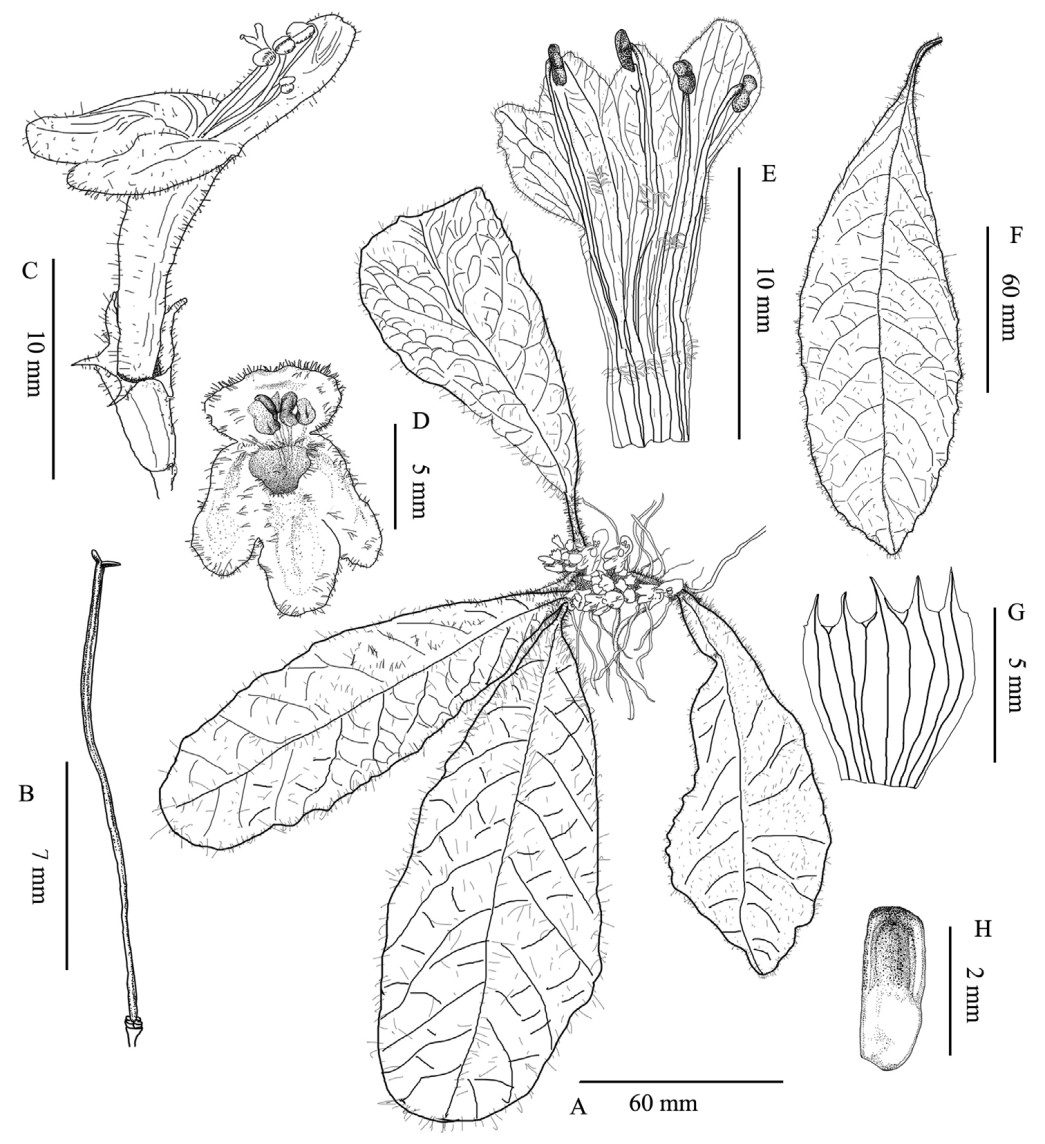
*Paraphlomis kuankuoshuiensis* sp. nov. **A** flowering plant **B** pistil **C** flower **D** front view of corolla **E** opened corolla **F** leaf **G** opened calyx **H** nutlet. Drawn by Tan Deng.

**Figure 3.**
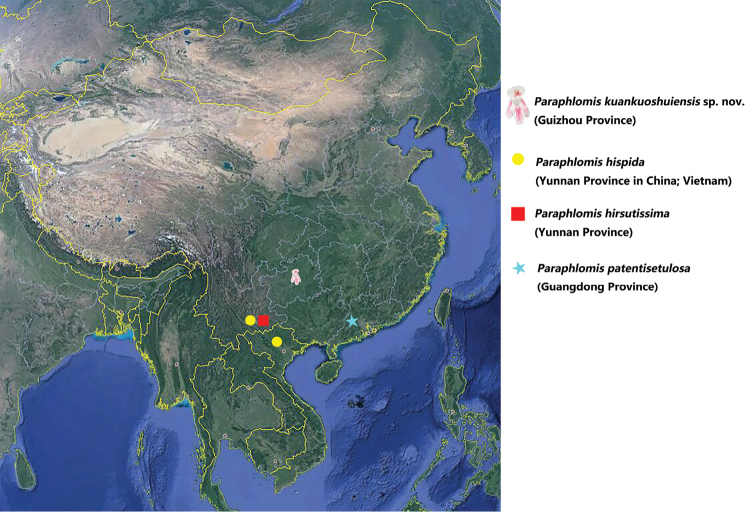
The geographical distribution of *Paraphlomis kuankuoshuiensis* sp. nov. and its three closely related species.

#### Type.

CHINA. Guizhou Province, Zunyi City, Suiyang County, Kuankuoshui National Natural Reserve, on moist rocks, 28°11'N, 107°04'E, 820 m alt., 22 July 2019, *ZRB1509* (fl., holotype ZY!, isotype IBK!), 24 August 2019 *ZRB1575* (fr., paratype ZY!).

#### Description.

Perennial herb. ***Rhizomes*** short, 2–4 cm, dense and fibrous roots. ***Stems*** 2–5 (–7), unbranched, 2–7 cm, slightly grooved, densely strigose, with 1 or 2 pairs of leaves for each stem. ***Leaves*** long elliptic or long obovate, (thickly) papery, 10–37 × 3–8 cm, apex obtuse or acute, base cuneate, margin serrulate; petioles 0.5–4 cm, adaxially slightly grooved, strigose; lateral veins in 8–12 pairs, obviously concave above and slightly raised below; adaxial surfaces and abaxial veins densely strigose. ***Inflorescence*** with one to two verticillasters; verticillasters 7-46-flowered; flowers shortly petiolate; apical opposite cymes globose, pseudoterminal; bracteoles linear-lanceolate, ca. 5 mm, margin ciliolate. ***Calyx*** tubular-campaniform, red; tube 5–6 mm, sparsely bristled, 10 veins; 5 teeth, unequal, triangular-lanceolate, 1–2 mm. ***Corolla*** white, 2-lipped, ca. 2.2 cm; tube obliquely hairy annulate inside; upper lip oblong, entire, galeate, with pink spots outside; lower lip 3-lobed, with a pink-striped interior and larger middle lobe. ***Stamens*** 4, anterior pair longer, all rising under upper corolla lip; filaments puberulent; anthers two-loculed, forked. ***Style*** filiform, exceeding stamens, apex 2-lobed, lobes subequal. ***Ovary*** 4-loculed, small ovary apex truncate, glabrous. ***Disc*** ring like, not obvious. **Nutlets** ca. 2.5 mm long, apex truncate, base attenuate. ***Fl.*** Jul–Aug. ***Fr.*** Aug–Sep.

#### Distribution and habitat.

Based on current field observations, *P. kuankuoshuiensis* is only located in the Dazhuxi and Matixi valleys, the Kuankuoshui National Natural Reserve, Suiyang County, Guizhou Province. The area has a subtropical monsoon climate and it is wet but not seasonly dry. It grows on moist steep limestone rocks (almost bare) beside streams at an altitude of approximately 820 m, and in groups of several thousand individuals.

#### Conservation status.

This species is currently known to only occur in two valleys, with a population numbering several thousand individuals. It is suggested it be placed in the Near Threatened IUCN category (IUCN 2017).

#### Phenology.

This new species was observed flowering from July to August and fruiting from August to September.

#### Etymology.

The specific epithet ‘*kuankuoshuiensis*’ is derived from the plant’s locality: Kuankuoshui National Natural Reserve, Guizhou Province, China.

## Supplementary Material

XML Treatment for Paraphlomis kuankuoshuiensis

## Supplementary materials

Supplementary material 1 Table S1. Digital *Paraphlomis* specimens checked at herbaria through NSII platform Data type: species data https://binary.pensoft.net/file/371994This dataset is made available under the Open Database License (http://opendatacommons.org/licenses/odbl/1.0/). The Open Database License (ODbL) is a license agreement intended to allow users to freely share, modify, and use this Dataset while maintaining this same freedom for others, provided that the original source and author(s) are credited.Ren-Bo Zhang, Tan Deng, Quan-Li Dou, Ruo-Xun Wei, Lin He, Chong-Bo Ma, Sheng Zhao, Shun Hu
