# Supplementary material for: Paraphlomis kuankuoshuiensis (Lamiaceae), a new species from the limestone areas of northern Guizhou, China
Source: PhytoKeys. 2020 Jan 15;139:13–20. doi: 10.3897/phytokeys.139.47055 (PMC6976690; doi:10.3897/phytokeys.139.47055)
Supplement: Supplementary material 1 [file phytokeys-139-013-s001.doc]

Table S1. Digital *Paraphlomis* specimens checked at herbaria through NSII platform.

| Taxon | Collecting no. | Herbarium |
| --- | --- | --- |
| *Paraphlomis albida* | 62441 | IBK |
| *Paraphlomis albida* | 144 | AU |
| *Paraphlomis albiflora* | 809 | SM |
| *Paraphlomis albiflora* | 0884 | SM |
| *Paraphlomis albotomentosa* | 080716087 | JIU |
| *Paraphlomis albotomentosa* | zdg1117 | JIU |
| *Paraphlomis foliata* | 831558 | FJFC |
| *Paraphlomis foliata* | 4708 | IBK |
| *Paraphlomis gracilis* | 4331221606290485LY | JIU |
| *Paraphlomis hirsutissima* | HGW-00147 | KUN |
| *Paraphlomis hirsutissima* | 10331 | NAS |
| *Paraphlomis hispida* | 92449 | IBK |
| *Paraphlomis hispida* | 3820 | BNU |
| *Paraphlomis intermedia* | 13268 | BH |
| *Paraphlomis intermedia* | 801228 | FJFC |
| *Paraphlomis javanica* | 73247 | IBK |
| *Paraphlomis javanica* | 41096 | IBK |
| *Paraphlomis kwangtungensis* | 48644 | IBK |
| *Paraphlomis lanceolata* | 450226160809080LY | GXMG |
| *Paraphlomis lanceolata* | 4331261409060573 | JIU |
| *Paraphlomis lancidentata* | 17242 | JJF |
| *Paraphlomis membranacea* | 9961 | KUN |
| *Paraphlomis membranacea* | 410 | KUN |
| *Paraphlomis pagantha* | 73217 | IBK |
| *Paraphlomis pagantha* | 27154 | IBK |
| *Paraphlomis paucisetosa* | 7185 | GXMG |
| *Paraphlomis paucisetosa* | 10554 | GXMG |
| *Paraphlomis reflexa* | 6708 | LBG |
| *Paraphlomis seticalyx* | 148852 | NAS |
| *Paraphlomis seticalyx* | 400943 | IBK |
| *Paraphlomis setulosa* | 830234 | IBK |
| *Paraphlomis subcoriacea* | 263 | IBK |
| *Paraphlomis subcoriacea* | 83710 | IBK |
